# Obesity during childhood is associated with higher cancer mortality rate during adulthood: the i3C Consortium

**DOI:** 10.1038/s41366-021-01000-3

**Published:** 2021-11-02

**Authors:** Joel Nuotio, Tomi T. Laitinen, Alan R. Sinaiko, Jessica G. Woo, Elaine M. Urbina, David R. Jacobs, Julia Steinberger, Ronald J. Prineas, Matthew A. Sabin, David P. Burgner, Heikki Minn, Trudy L. Burns, Lydia A. Bazzano, Alison J. Venn, Jorma S. A. Viikari, Nina Hutri-Kähönen, Stephen R. Daniels, Olli T. Raitakari, Costan G. Magnussen, Markus Juonala, Terence Dwyer

**Affiliations:** 1 Research Centre of Applied and Preventive Cardiovascular Medicine, University of Turku, Turku, Finland; 2 Heart Center, Turku University Hospital and University of Turku, Turku, Finland; 3 Murdoch Children’s Research Institute, Melbourne, VIC Australia; 4 Centre for Population Health Research, University of Turku and Turku University Hospital, Turku, Finland; 5 Paavo Nurmi Centre, Sports and Exercise Medicine Unit, Department of Physical Activity and Health, University of Turku, Turku, Finland; 6 Department of Pediatrics, University of Minnesota School of Medicine, Minneapolis, MN USA; 7 Department of Pediatrics, University of Cincinnati College of Medicine, Cincinnati, OH USA; 8 Division of Biostatistics and Epidemiology, Cincinnati Children’s Hospital Medical Center, Cincinnati, OH USA; 9 The Heart Institute, Cincinnati Children’s Hospital Medical Center, Cincinnati, OH USA; 10 Division of Epidemiology and Community Health, School of Public Health, University of Minnesota, Minneapolis, MN USA; 11 Division of Public Health Science, Wake Forest University, Winston-Salem, NC USA; 12 Department of Paediatrics, The University of Melbourne, Parkville, VIC Australia; 13 Department of Endocrinology and Diabetes, The Royal Children’s Hospital, Parkville, VIC Australia; 14 Department of Oncology, Turku University Hospital, Turku, Finland; 15 Department of Epidemiology, College of Public Health, University of Iowa, Iowa City, IA USA; 16 Department of Epidemiology, Tulane University School of Public Health and Tropical Medicine, New Orleans, LA USA; 17 Menzies Institute for Medical Research, University of Tasmania, Hobart, TAS Australia; 18 Department of Internal Medicine, University of Turku, Turku, Finland; 19 Division of Medicine, Turku University Hospital, Turku, Finland; 20 Department of Pediatrics, Tampere University Hospital, and Faculty of Medicine and Health Technology, Tampere University, Tampere, Finland; 21 Department of Clinical Physiology and Nuclear Medicine, Turku University Hospital, Turku, Finland; 22 Oxford Martin School, Oxford University, Oxford, UK

**Keywords:** Obesity, Risk factors, Cancer

## Abstract

**Background:**

In high-income countries, cancer is the leading cause of death among middle-aged adults. Prospective data on the effects of childhood risk exposures on subsequent cancer mortality are scarce.

**Methods:**

We examined whether childhood body mass index (BMI), blood pressure, glucose and lipid levels were associated with adult cancer mortality, using data from 21,012 children enrolled aged 3–19 years in seven prospective cohort studies from the U.S., Australia, and Finland that have followed participants from childhood into adulthood. Cancer mortality (cancer as a primary or secondary cause of death) was captured using registries.

**Results:**

354 cancer deaths occurred over the follow-up. In age-, sex, and cohort-adjusted analyses, childhood BMI (Hazard ratio [HR], 1.13; 95% confidence interval [CI] 1.03–1.24 per 1-SD increase) and childhood glucose (HR 1.22; 95%CI 1.01–1.47 per 1-SD increase), were associated with subsequent cancer mortality. In a multivariable analysis adjusted for age, sex, cohort, and childhood measures of fasting glucose, total cholesterol, triglycerides, and systolic blood pressure, childhood BMI remained as an independent predictor of subsequent cancer mortality (HR, 1.24; 95%CI, 1.03–1.49). The association of childhood BMI and subsequent cancer mortality persisted after adjustment for adulthood BMI (HR for childhood BMI, 1.35; 95%CI 1.12–1.63).

**Conclusions:**

Higher childhood BMI was independently associated with increased overall cancer mortality.

## Introduction

In high-income countries, cancer is the leading cause of mortality among middle-aged adults [1]. In adulthood, obesity has been shown to increase the risk for numerous cancers and as globally more people are overweight than are underweight this substantially contributes to the cancer-related burden [2, 3]. Nonetheless, exposure to established modifiable cancer risk factors in adulthood, such as obesity, usually are manifest in childhood. Exposures commencing in childhood and adolescence may be especially harmful if they increase cumulative lifetime exposure or if they act during critical developmental periods [4, 5].

To date, epidemiologic research on risk factors for cancer has focused primarily on older adult populations; very little is known about the etiological relevance of exposure to modifiable risk factors in earlier stages of the life course [4, 6]. In a recent study, the risk of developing an obesity-related cancer was shown to increase in a stepwise manner in successively younger birth cohorts in the U.S [7]. A Danish study based on health records demonstrated that childhood overweight persisting into young adulthood was associated with increased risk for esophageal, gastric and colon cancer in men [8, 9]. In addition, results from a prospective study among American Indians suggested that obesity, hypertension, and glucose intolerance in childhood were major predictors of premature death from endogenous causes [10]. However, little is known about the relationship between these childhood risk factors and subsequent cancer mortality.

In this study, we used data among 21,012 individuals from seven prospective cohort studies from the U.S., Australia, and Finland that have followed participants from childhood into adulthood. Our main aim was to examine whether childhood body mass index (BMI), blood pressure, glucose and lipid levels are associated with subsequent cancer mortality.

## Materials and methods

### Study sample

**Fig. 1:**
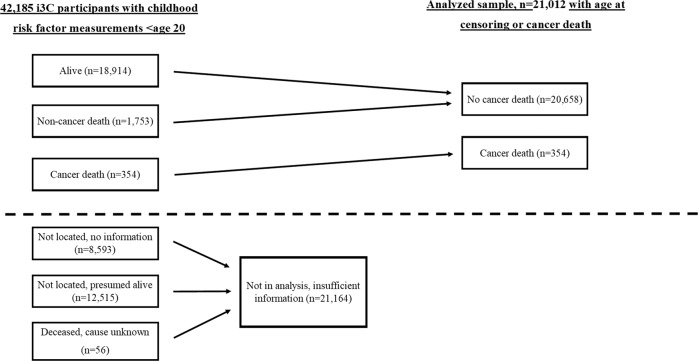
Flow diagram of the 42,185 i3C Consortium participants with any childhood risk factor measurement prior to age 20 and participants with data available on outcomes (analyzed sample).

The sample comprised 21,012 participants aged 3–19 years at baseline from the International Childhood Cardiovascular Cohort (i3C) Consortium, in seven childhood cohorts from the U.S., Australia, and Finland (Fig. 1) [11, 12]. All seven cohorts included in this analysis have been previously described in detail [11], and a brief description follows. The cohort studies followed protocols approved by local ethics committees, with signed informed consent. All cohorts had data collected in clinical examinations that obtained the participants’ age, sex, height and weight, and blood pressure. All laboratory measurements were performed using fasting blood samples. First available measure of childhood risk factors was used in the analyses. Data on childhood smoking was assessed with questionnaires and participants who had smoked daily during childhood were classified as smokers. Information on childhood family socioeconomic status was obtained with questionnaires and information on maximum parental education classified into four categories (1 = less than high school, 2 = high school or equivalent, 3 = more than high school or equivalent including some college studies but not a college degree, 4 = university degree).

### The Bogalusa heart study

The Bogalusa heart study (BHS) began in 1973, with nine subsequent cross-sectional screenings of children aged 3 to 18 years and participation rates of 80–93%. An additional 11 cross-sectional adulthood screenings at ages 19–62 years were conducted, with participation rates of 72 to 93%. Linking the 20 surveys provides 39,647 observations on 11,961 cohort members. In the most recent contact 1298 of a subsample of recruited participants was examined between 2013 and 2016.

### Minneapolis childhood cohort studies

The Minneapolis Childhood Cohort Studies (Minnesota) consist of three separate cohort studies, with recruitment during childhood and with repeated examinations into the third-fourth decades. Altogether, a total of 1817 participants have been followed longitudinally in the Minneapolis studies, beginning at age 7–14; of these, repeat examination data are available for 1300 participants aged ≥ 25 years.

### The Muscatine study

The Muscatine study (Muscatine) was initiated in 1970 in the schools of Muscatine, Iowa. Approximately 70% of the eligible school population (a total of 11,377 students, 5–18 years at baseline) had data collected up to six times over the following 12 years. During 1982–1991, a representative subsample of the school-based cohort was re-examined one or two times during young adulthood (*N* = 2547 at ages ranging from 20 to 39 years), and a representative subsample of the young adults (*N* = 906) was followed longitudinally during middle adulthood with up to nine clinic examinations between 1992 and 2008. A subset (*N* = 594) of the adult cohort was recruited in 1999–2001 for an examination, and the most recent follow-up examination of this subset was conducted in 2007–2008 with 82% participation.

### The Princeton Lipid Research Study

The Princeton Lipid Research Study (PLRS) began in 1973 as part of the National Heart, Lung, and Blood Institute (NHLBI) Lipid Research Clinics Prevalence Study with enrollment of 6775, 6–19-year old children from the Princeton City School District (Cincinnati, OH). A random subset of 2324 participants was selected for two childhood visits, and a family-based subset of those was seen between 1998 and 2003 and participated in a heath-status questionnaire in 2010–2012. The last in-person examination was conducted between 1998 and 2001 on 911 participants, 70% of those eligible for recruitment.

### The NHLBI growth and health study

The NHLBI Growth and Health Study (NGHS) was started in 1987 as a longitudinal fixed-cohort study of girls (9–10 years at baseline and followed annually for 10 years to age 19) at three clinical centers (Richmond, CA, Cincinnati, OH, and Washington, DC). Only the Cincinnati center (*N* = 870) is participating in this study. Additional yearly examinations were conducted at the Cincinnati center between ages 19–24, and there were two visits between ages 25–29. The last in-person examination was conducted between 2003 and 2006, with recruitment of 535 participants, 70% of those eligible.

### The childhood determinants of adult health study

The Childhood Determinants of Adult Health (CDAH) Study started in 1985 with recruitment across Australia of 8498 school children aged 7–15. Technical tests and blood samples were collected on 9-, 12-, and 15-year old children. Clinical follow-up studies were conducted in 2004–2006 and 2014–2019, and in 2009–2010, a questionnaire-based assessment of health status was obtained.

### The Cardiovascular risk in young finns study

The Cardiovascular Risk in Young Finns Study (YFS) is a population-based multicenter study in Finland and the largest European CV risk factor follow-up study from childhood to adulthood. Participants aged 3–18 years (*N* = 3596) were recruited in 1980, and this cohort has been followed-up every 3–6 years. The most recent in-person examination was conducted in 2011 with participation of 2,063 individuals (57% of the original study population).

### Outcome: cancer mortality

Searches for deceased cohort members and causes of death were conducted in all cohorts in years 2016–2018. The International Statistical Classification of Diseases and Related Health Problems codes (8th, 9th, and 10th revisions; ICD-8, ICD-9 and ICD-10, respectively) used in the search were 140.0–209.9 (ICD-8 and ICD-9) and C00.0-C97.9 (ICD-10). In the U.S., searches were conducted via the U.S. National Death Index (NDI), a database of all deaths in the U.S. since 1979. Decedents’ cause of death were obtained from NDI coding, with death certificates obtained for validation when possible. The YFS and CDAH had access to the Finnish and Australian NDIs, respectively, to search for deceased participants and causes of death. Cancer as a primary (underlying) cause of death and cancer as a secondary (significant condition contributing to death but not underlying) cause of death were both classified as cancer deaths.

### Statistical analyses

Values are presented as mean and SD, or proportions. Values for triglycerides, BMI, and glucose were log-transformed due to skewness of distribution. Age group-, study cohort- and sex-specific z-scores were constructed for each exposure measure. To examine the associations of childhood exposures and subsequent cancer mortality, regression analyses of survival data based on the Cox proportional hazards model were used. Sex by risk factor interactions were studied to investigate if the associations with cancer mortality were similar in males and females. No interaction effects were observed (*P* value for interaction >0.20 in all univariate analyses), and therefore males and females were combined for analysis. As earlier studies have examined the relationship between childhood exposures and premature deaths using quartiles of childhood exposures [10, 13], a childhood risk score that assigned a value of 1 for childhood BMI > 75th percentile-point, and childhood fasting glucose > 75th percentile-point was formed for cohorts that had data available for both childhood BMI and childhood glucose (BHS, NGHS, Minnesota, PLRS, YFS). The range of the score was from 0 to 2 and age-, sex-, and cohort-specific percentile-points were used. Furthermore, previous studies have shown that childhood overweight persisting into young adulthood increased the risk for esophageal, gastric and colon cancer in men [8, 9]. Therefore, we examined the association of childhood overweight using age- and sex-specific cut-off points defined by the International Obesity Task Force [14] and both cancer mortality related to esophageal, gastric, and colorectal cancers and overall cancer mortality. Statistical analyses were performed with SAS 9.4. Statistical significance was inferred at a two-tailed value of *P* ≤ 0.05.

## Results

The study sample consisted of 21,012 participants (9740 males and 11,272 females) with a mean age of 9.9 ± 3.9 years at baseline. Characteristics of the participants according to cohort are shown in Table 1. There were altogether 354 cancer deaths among study participants (mean age at death 45.1 ± 12.1 years), of which 20 (5.7%) occurred during childhood (age < 20 years). All cancer-related deaths and specific diagnoses are shown in Supplementary Table 1. The most common cancer diagnoses in this study were malignant neoplasm of unspecified part of bronchus or lung (C34.9, 53 cases), malignant neoplasm of breast of unspecified site (C50.9, 31 cases), malignant unspecified neoplasm of colon (C18.9, 20 cases), malignant unspecified neoplasm of pancreas (C25.9, 19 cases), and malignant unspecified neoplasm of brain (C71.9, 19 cases).

**Table 1:** Characteristics of the study participants according to cohort.

		BHS (*N* = 4370)		CDAH (*N* = 3220)		MN (*N* = 1249)		MUSC (*N* = 7032)		NGHS (*N* = 515)		PLRS (*N* = 1033)			YFS (*N* = 3593)	
Sex (% female)		54.6		54.4		49.6		51.1		100		55.9			51.0	
Blacks (%)		43.9		0.7		16.7		0.6		45.8		23.2			0.0	
Age at baseline, y		8.7	(3.2)	11.1	(2.6)	9.8	(3.4)	9.9	(3.6)	9.4	(0.5)	12.2	(3.4)	10.4		(5.0)
Total cholesterol, mmol/L		4.30	(0.75)	4.52	(0.76)	3.93	(0.70)	4.09	(0.72)	4.36	(0.79)	4.46	(0.82)	5.29		(0.92)
LDL-C, mmol/L		2.46	(0.67)	2.74	(0.70)	2.29	(0.62)	2.36	(0.62)	2.63	(0.74)	2.73	(0.74)	3.43		(0.84)
Triglycerides, mmol/L		0.77	(0.38)	0.74	(0.40)	1.07	(0.63)	0.87	(0.44)	0.90	(0.37)	0.90	(0.46)	0.66		(0.32)
BMI, kg/m^2^		17.7	(3.6)	18.2	(2.8)	19.0	(4.7)	18.4	(3.7)	18.3	(3.7)	19.7	(4.3)	17.8		(3.1)
Systolic blood pressure, mmHg		99	(10)	109	(13)	106	(11)	108	(15)	101	(8)	104	(13)	112		(12)
Diastolic blood pressure, mmHg		45	(13)	66	(11)	58	(13)	64	(12)	59	(10)	63	(11)	68		(10)
Fasting glucose, mmol/mol		4.67	(0.71)	N/A		4.93	(0.48)	N/A		4.81	(1.08)	4.77	(0.55)	4.73		(0.89)
Daily smoking (%)		41.3		33.9		39.0		33.6		41.3		30.0		41.5		
Parental education (%)	< high school	11.8		27.6		8.3		11.2		7.6		20.4		52.23		
	= high school degree	38.8		11.2		35.4		44.6		25.8		34.0		12.41		
	> high school but no college degree	19.2		31.2		21.7		17.5		47.4		19.2		23.65		
	≥ college degree	30.3		30.0		34.6		26.8		19.2		26.5		11.7		
No. of cancer deaths		100		19		7		175		4		30			19	
Follow-up time, y, mean (range)		34.4	(1–47)	31.5	(1–35)	31.1	(0.3–42)	42.5	(1–50)	29.2	(13–33)	40.6	(3–46)	37.0		(1–38)

Values are mean (SD) unless otherwise stated.

*BHS* Bogalusa Heart Study, *CDAH* Childhood Determinants of Adult Health Study, *MN* Minnesota Childhood Cardiovascular Cohorts, *MUSC* Muscatine Study, *NGHS* NHLBI Growth and Health Study, *PLRS* Princeton Lipid Research Study, *YFS* Cardiovascular Risk in Young Finns Study, *LDL-C* low-density lipoprotein cholesterol, *HDL-C* high-density lipoprotein cholesterol, *BMI* body mass index.

### Associations between childhood risk factors and cancer mortality

Childhood BMI (HR 1.13; 95% CI 1.03–1.24 per 1-SD increase in childhood BMI) and fasting glucose (HR 1.22; 95% CI 1.01–1.47 per 1-SD increase in childhood fasting glucose) were associated with increased risk for subsequent cancer mortality in analyses adjusted for age, sex and study cohort (Table 2). No associations with overall cancer mortality were observed for childhood blood pressure, lipid levels or smoking. Because socioeconomic factors could be possible confounders for the observed associations, we further adjusted the analyses shown in Table 2 for childhood family socioeconomic status in a subsample that had these data available. After this adjustment, HR for both childhood BMI (HR 1.11; 95% CI 0.97–1.28) and childhood fasting glucose (HR 1.20; 95% CI 0.95–1.53) remained essentially similar but the confidence intervals slightly widened (Supplementary Table 2).

**Table 2:** Risk factors in childhood: association with subsequent cancer mortality.

	*n* (cancer deaths) / N (number of censored)	Hazard ratio*	95% CI*		*P*
Systolic blood pressure	335 / 18,583	1.02	0.91	1.13	0.79
Diastolic blood pressure	332 / 18,475	0.96	0.86	1.07	0.49
Body mass index	350 / 20,954	1.13	1.03	1.24	0.01
LDL-cholesterol	167 / 11,873	1.01	0.86	1.18	0.93
Total cholesterol	324 / 16,772	1.00	0.89	1.11	0.94
Triglycerides	322/16,629	1.03	0.92	1.15	0.65
Smoking	193/19,510	1.04	0.78	1.39	0.78
Glucose	116 / 7,805	1.22	1.01	1.47	0.04

Values for body mass index, triglycerides, and glucose were log-transformed due to skewness of distribution. Continuous variables were standardized according to age, sex, and cohort. Models were adjusted for age, sex, and cohort. * Per one standard deviation increase.

The association of childhood BMI and subsequent cancer mortality remained significant after further concurrent adjustments for childhood measures of fasting glucose, systolic blood pressure, triglycerides, and total cholesterol (HR 1.24; 95% CI 1.03–1.49). In this multivariable analysis, the association of childhood fasting glucose and subsequent cancer mortality was similar but not statistically significant (HR 1.19; 95% CI 0.98–1.19) (Table 3). The results remained similar after additional adjustment for ethnicity (HR 1.21; 95% CI 1.01–1.45 for BMI and HR 1.19; 95% CI 0.99–1.44 for fasting glucose).

**Table 3:** Multivariable model examining the association of childhood risk factors and subsequent cancer mortality (*N* = 7405, number of cancer deaths = 113).

	Hazard ratio*	95% CI*		*P*
Systolic blood pressure	0.93	0.76	1.13	0.47
Body mass index	1.24	1.03	1.49	0.02
Triglycerides	0.93	0.76	1.14	0.49
Total cholesterol	1.01	0.83	1.23	0.96
Glucose	1.19	0.98	1.19	0.08

Continuous variables were standardized according to age, sex and cohort. The analysis was additionally adjusted for age, sex, and cohort.

*Per one standard deviation increase.

**Fig. 2:**
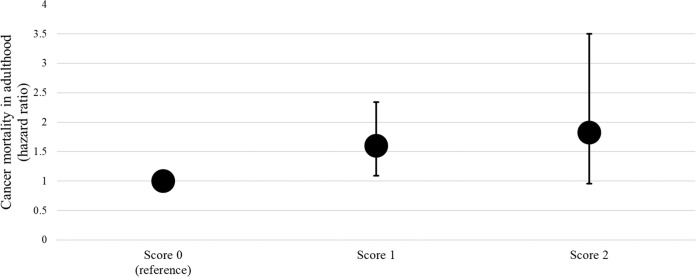
Risk of cancer mortality according to childhood risk points. Childhood risk points: BMI and/or glucose >75th age, sex, and study-specific percentile point. Analysis adjusted for age, sex, and study cohort. Cohorts included in this analysis were BHS, NGHS, Minnesota, PLRS, and YFS that had glucose data available.

To further explore the observed associations between childhood BMI and fasting glucose and cancer mortality, we first performed analyses additionally adjusted for adult BMI. In this analysis, the relationship between childhood BMI and cancer mortality remained essentially similar (HR for childhood BMI, 1.35; 95% CI 1.12–1.63). Second, we examined the associations in different countries separately. In these analyses, childhood BMI was associated with subsequent cancer mortality in the U.S. cohorts and in the Australian cohort. Childhood fasting glucose (data not available in the Australian cohort) was associated with subsequent cancer mortality in the U.S. cohorts (Table 4). Third, we created a childhood risk score that assigned a value of 1 for childhood BMI > 75th age-, sex-, and cohort-specific percentile, and childhood fasting glucose >75th age-, sex-, and cohort-specific percentile (possible score range 0–2). The childhood risk score was directly associated with subsequent cancer mortality (*P* = 0.028). Furthermore, children with only one of these two risk factors had an increased risk for subsequent cancer mortality (*P* = 0.016) (Fig. 2). Fourth, we examined the relationships between childhood overweight defined by age- and sex-specific cut-off points [14] and both overall cancer mortality and cancer mortality due to esophageal, gastric and colorectal cancers. Childhood overweight was associated with overall cancer mortality (HR 1.49 (95%CI 1.17–1.90)). No association between childhood overweight and esophageal, gastric and colorectal cancer-related deaths was observed (HR 0.78; 95%CI 0.35–1.75). Fifth, we compared the risk of subsequent cancer mortality between participants with childhood BMI > 95th percentile and childhood BMI ≤ 95th percentile. In this analysis, participants with childhood BMI > 95th percentile had a higher risk for overall cancer mortality compared to those with BMI ≤ 95th percentile (HR 1.72; 95% CI 1.15–2.56).

**Table 4:** Association of childhood body mass index and glucose and subsequent cancer mortality stratified by country.

	Body mass index					Glucose				
	n/N*	Hazard ratio**	95% CI**		*P*	n/N*	Hazard ratio**	95% CI**		*P*
CDAH	19/3,200	1.53	1.11	2.13	0.01	N/A				
Young Finns Study	19/3,572	0.99	0.63	1.55	0.96	6/1,802	1.18	0.67	2.08	0.58
U.S. cohorts***	312/13,832	1.11	1.00	1.24	0.04	110/5,887	1.23	1.01	1.49	0.04

Analyses adjusted for age and sex and study cohort.

*n (cancer deaths) / N (number of censored).

** Per one standard deviation increase.

***U.S. cohorts included BHS, NGHS, Minnesota, and PLRS with data on childhood glucose available.

## Discussion

This study of seven international cohorts examined show that high childhood BMI and high fasting glucose concentration were associated with subsequent cancer mortality. The association of childhood BMI and subsequent cancer mortality was independent of other examined childhood risk factors. Importantly, the effect of childhood BMI on cancer mortality persisted after adjustment for adulthood BMI.

Building upon the substantial data on adult adiposity and cancer, some previous observational studies have related adiposity in adolescence to subsequent malignancies such as leukemia, Hodgkin’s lymphoma, colorectal cancer, breast cancer and others in adulthood [3, 15–19]. The main advantage of the present study over the previous reports is that we were able to take into account the role of several other possible childhood risk factors of subsequent cancer mortality and examine the association prospectively in seven large international cohorts from three continents. In addition, we specifically examined the association with cancer mortality. In the present study, childhood BMI was associated with increased risk of cancer mortality independently of childhood measures of fasting glucose, total cholesterol, triglycerides, and systolic blood pressure. Further, a novel finding of this study is that the effect of childhood BMI on subsequent cancer mortality was not explained by adulthood BMI, suggesting that childhood BMI may be an independent risk factor for subsequent cancer mortality.

In this study, the most frequent cancer-related deaths were due to lung cancer, breast cancer and colon cancer, which are all also listed among the five most common causes of cancer deaths by the WHO [20]. As the number of diagnosis-specific cancer deaths was relatively low in the present study, we examined the relationship of childhood exposures and subsequent overall cancer mortality. Earlier studies have shown that childhood overweight persisting into young adulthood increases the risk for adult esophageal, gastric and colorectal cancer [8, 9]. Therefore, we examined the relationship of childhood overweight defined by age- and sex-specific cut-off points [14] and these diagnosis-specific cancer-related deaths, but no association was observed. This analysis might be underpowered due to the relatively low number of esophageal, gastric and colorectal cancer deaths (*N* = 46). However, childhood overweight defined by these cut-off points was associated with overall cancer mortality.

To the best of our knowledge, this study is the first to report an association between childhood fasting glucose and subsequent cancer mortality. Previously it has been shown that glucose intolerance in childhood strongly associates with increased rates of premature death from endogenous causes [10]. In addition, a recent report showed that the number of cancer-related deaths is higher among people with diabetes compared to individuals without diabetes [21]. Therefore, one possible explanation for the findings of the present study is that higher glucose levels already during childhood and adolescence may impact cancer mortality risk by increasing the duration and lifetime exposure to higher blood glucose levels and associated comorbidities and/or by acting during critical developmental periods. It is also possible that the association between elevated fasting glucose and higher cancer mortality risk may not be causative per se, but may be triggered by other risk markers of the metabolic syndrome, which in turn mediates a subclinical inflammatory response already early in life [22].

Childhood BMI is a predictor of adult BMI, and obesity is difficult to manage once it is established [23]. Importantly, in this study we observed that children with higher BMI had an increased risk for cancer mortality, independently of their BMI in adulthood. Early life exposure to higher BMI increases the risk and severity of insulin resistance, oxidative DNA damage, chronic inflammation, and results in changes to endogenous hormone metabolism, which are all plausible contributory mechanisms linking obesity and cancer [24]. Although the observational nature of our study precludes making clinical recommendations, we hypothesize that primordial prevention of obesity in children and adolescents could reduce their risk for subsequent cancer mortality. Thus, childhood and adolescence, a period essential for determining exposures over the life-course, may be an optimal period to intervene to lower subsequent cancer risk.

The main strength of this study is the use of pooled data on childhood risk factors and adult cancer mortality from seven international longitudinal cohorts. However, the study has some potential limitations. First, even though we were able to follow up the participants until middle age, the follow-up time may still be too short to show the total impact of childhood BMI and other risk factors on cancer mortality since many cancers are first detected among elderly people. Second, there was a relatively large variety in the proportion of black participants among the study cohorts (between 0% in the YFS and 46% in the NGHS), which may have led to some bias. However, the main results remained similar after adjustment for ethnicity. Third, data on childhood glucose were not available from two of the seven cohorts included in this study. Fourth, cohort studies, which are observational, are prone to some forms of bias, notably due to loss to follow up and confounding. In this study loss to follow up is minimal as deaths are completely recorded in the countries that were involved in the i3C Consortium. We accounted for those confounding factors we could by adjusting for them in the analysis. There may, nonetheless, be unmeasured confounding. Finally, we did not have data available on cancer morbidity or the success of cancer treatments but only on cancer-related deaths. Therefore, we were not able to differentiate whether excess bodyweight or other participant characteristics in childhood or adulthood were associated with increased cancer incidence and/or associated with higher mortality among cancer patients. Previous studies in adults have shown that obesity is associated with both higher cancer incidence and worse survival supporting both explanations [25–28].

## Conclusion

We observed that high childhood BMI was independently associated with subsequent cancer mortality. Primordial prevention of overweight and obesity in children and adolescents should be emphasized to reverse the obesity epidemic and thereby possibly reduce the burden of cancer mortality.

## Supplementary information

Supplemental material
