# Supplementary material for: Obesity during childhood is associated with higher cancer mortality rate during adulthood: the i3C Consortium
Source: Int J Obes (Lond). 2021 Nov 2;46(2):393–9. doi: 10.1038/s41366-021-01000-3 (PMC8794778; doi:10.1038/s41366-021-01000-3)
Supplement: Supplementary file 1 — Supplemental material [file 41366_2021_1000_MOESM1_ESM.docx]

**Supplementary Material. Childhood risk factors predicting cancer mortality: The i3C Consortium**

**Supplemental Table 1.** The number of cancer diagnoses in Australia, Finland and the U.S. according to the International Statistical Classification of Diseases and Related Health Problems codes.

| **ICD-8** | | | | |
| --- | --- | --- | --- | --- |
| Diagnosis | | Australia | Finland | US |
| 171.1 | Malignant neoplasm of trunk | 0 | 1 | 0 |
| 202.2 | Other primary malignant neoplasms of lymphoid tissue | 0 | 1 | 0 |
| 204.9 | Lymphatic leukaemia, unspecified | 0 | 1 | 0 |
| 204.0 | Acute lymphoid leukemia | 0 | 1 | 0 |
| 205.0 | Acute myeloid leukemia | 0 | 1 | 0 |
| **ICD-9** | | | | |
| Diagnosis | | Australia | Finland | US |
| 141.9 | Malignant neoplasm of tongue, unspecified | 0 | 0 | 1 |
| 151.4 | Malignant neoplasm of body of stomach | 1 | 0 | 0 |
| 151.9 | Malignant neoplasm of stomach, unspecified site | 0 | 0 | 1 |
| 153.9 | Malignant neoplasm of colon, unspecified site | 0 | 0 | 2 |
| 1550 | Malignant neoplasm of liver, primary | 0 | 0 | 1 |
| 160.2 | Malignant neoplasm of maxillary sinus | 0 | 0 | 1 |
| 162.9 | Malignant neoplasm of bronchus and lung, unspecified | 0 | 0 | 2 |
| 170.9 | Malignant neoplasm of bone and articular cartilage, site unspecified | 0 | 0 | 1 |
| 171.9 | Malignant neoplasm of connective and other soft tissue, site unspecified | 0 | 0 | 3 |
| 172.9 | Melanoma of skin, site unspecified | 0 | 0 | 1 |
| 174.9 | Malignant neoplasm of breast (female), unspecified | 0 | 0 | 2 |
| 179.0 | Malignant neoplasm of uterus, part unspecified | 0 | 0 | 1 |
| 180.9 | Malignant neoplasm of cervix uteri, unspecified site | 0 | 0 | 2 |
| 191.0 | Malignant neoplasm of cerebrum, except lobes and ventricles | 1 | 0 | 0 |
| 191.5 | Malignant neoplasm of ventricles | 0 | 0 | 1 |
| 191.7 | Malignant neoplasm of brain stem | 0 | 0 | 1 |
| 191.9 | Malignant neoplasm of brain, unspecified | 0 | 0 | 5 |
| 197.0 | Secondary malignant neoplasm of lung | 0 | 0 | 1 |
| 197.6 | Secondary malignant neoplasm of retroperitoneum and peritoneum | 0 | 0 | 1 |
| 197.7 | Malignant neoplasm of liver, secondary | 0 | 0 | 1 |
| 198.3 | Secondary malignant neoplasm of brain and spinal cord | 0 | 0 | 3 |
| 198.5 | Secondary malignant neoplasm of bone and bone marrow | 0 | 0 | 1 |
| 198.8 | Secondary malignant neoplasm of other specified sites | 0 | 0 | 1 |
| 199.0 | Disseminated malignant neoplasm without specification of site | 0 | 0 | 1 |
| 199.1 | Other malignant neoplasm without specification of site | 0 | 0 | 3 |
| 201.0 | Hodgkin's paragranuloma | 0 | 0 | 1 |
| 201.9 | Hodgkin's disease unspecified type | 0 | 0 | 2 |
| 202.8 | Lymphoma (malignant) | 0 | 0 | 5 |
| 203.8 | Other immunoproliferative neoplasms | 0 | 0 | 1 |
| 204.0 | Acute lymphoblastic leukemia | 1 | 0 | 2 |
| 205.0 | Acute myelogenous leukemia | 0 | 0 | 4 |
| 208.0 | Leukemia of unspecified cell type | 0 | 0 | 1 |
| **ICD-10** | | | | |
| Diagnosis | | Australia | Finland | US |
| C02.9 | Malignant neoplasm of tongue, unspecified | 0 | 0 | 2 |
| C06.9 | Malignant neoplasm of mouth, unspecified | 0 | 0 | 1 |
| C07 | Malignant neoplasm of parotid gland | 0 | 0 | 1 |
| C09.9 | Malignant neoplasm of tonsil, unspecified | 0 | 0 | 2 |
| C10.9 | Malignant neoplasm of oropharynx, unspecified | 0 | 0 | 2 |
| C13.9 | Malignant neoplasm of hypopharynx, unspecified | 0 | 0 | 1 |
| C14.0 | Malignant neoplasm of pharynx, unspecified | 0 | 0 | 1 |
| C15.5 | Malignant neoplasm of lower third of esophagus | 0 | 0 | 1 |
| C15.9 | Malignant neoplasm of esophagus, unspecified | 1 | 0 | 9 |
| C16.9 | Malignant neoplasm of stomach, unspecified | 0 | 0 | 4 |
| C18.1 | Malignant neoplasm of appendix | 0 | 0 | 1 |
| C18.7 | Malignant neoplasm of sigmoid colon | 0 | 1 | 1 |
| C18.9 | Malignant neoplasm of colon, unspecified | 0 | 0 | 20 |
| C19 | Malignant neoplasm of rectosigmoid junction | 0 | 0 | 3 |
| C20 | Malignant neoplasm of rectum | 0 | 0 | 1 |
| C21.1 | Malignant neoplasm of anal canal | 0 | 0 | 1 |
| C22.0 | Liver cell carcinoma | 0 | 0 | 9 |
| C22.1 | Intrahepatic bile duct carcinoma | 0 | 0 | 2 |
| C22.9 | Malignant neoplasm of liver, not specified as primary or secondary | 0 | 0 | 3 |
| C23 | Malignant neoplasm of gallbladder | 0 | 0 | 1 |
| C25.0 | Malignant neoplasm of head of pancreas | 0 | 1 | 0 |
| C25.9 | Malignant neoplasm of pancreas, unspecified | 1 | 0 | 18 |
| C26.9 | Malignant neoplasm of ill-defined sites within the digestive system | 0 | 0 | 1 |
| C32.9 | Malignant neoplasm of larynx, unspecified | 0 | 0 | 2 |
| C34.1 | Malignant neoplasm of upper lobe, bronchus or lung | 0 | 0 | 1 |
| C34.9 | Malignant neoplasm of unspecified part of bronchus or lung | 2 | 1 | 50 |
| C41.9 | Malignant neoplasm of bone and articular cartilage, unspecified | 1 | 0 | 0 |
| C43.3 | Malignant melanoma of other and unspecified parts of face | 1 | 0 | 0 |
| C43.6 | Malignant melanoma of upper limb, including shoulder | 0 | 1 | 1 |
| C43.7 | Malignant melanoma of lower limb, including hip | 0 | 0 | 0 |
| C43.9 | Malignant melanoma of skin, unspecified | 2 | 0 | 6 |
| C44.3 | Other and unspecified malignant neoplasm of skin of other and unspecified parts of face | 0 | 0 | 1 |
| C49.2 | Malignant neoplasm of connective and soft tissue of lower limb, including hip | 0 | 0 | 1 |
| C49.9 | Malignant neoplasm of connective and soft tissue, unspecified | 1 | 0 | 2 |
| C50.1 | Malignant neoplasm of central portion of breast | 0 | 1 | 0 |
| C50.8 | Malignant neoplasm of overlapping sites of breast | 0 | 1 | 0 |
| C50.9 | Malignant neoplasm of breast of unspecified site | 2 | 2 | 27 |
| C51.9 | Malignant neoplasm of vulva, unspecified | 0 | 0 | 1 |
| C53.0 | Malignant neoplasm of endocervix | 0 | 0 | 1 |
| C53.9 | Malignant neoplasm of cervix uteri, unspecified | 0 | 0 | 3 |
| C54.1 | Malignant neoplasm of endometrium | 0 | 0 | 3 |
| C54.9 | Malignant neoplasm of corpus uteri, unspecified | 1 | 0 | 0 |
| C55 | Malignant neoplasm of uterus, part unspecified | 0 | 0 | 1 |
| C56 | Malignant neoplasm of ovary | 0 | 0 | 8 |
| C57.0 | Malignant neoplasm of fallopian tube | 0 | 0 | 1 |
| C61 | Malignant neoplasm of prostate | 0 | 0 | 4 |
| C62.9 | Malignant neoplasm of testis, unspecified whether descended or undescended | 0 | 0 | 1 |
| C64 | Malignant neoplasm of kidney, except renal pelvis | 0 | 0 | 8 |
| C67.9 | Malignant neoplasm of bladder, unspecified | 0 | 0 | 3 |
| C71.2 | Malignant neoplasm of temporal lobe | 2 | 0 | 0 |
| C71.3 | Malignant neoplasm of parietal lobe | 0 | 1 | 0 |
| C71.7 | Malignant neoplasm of brain stem | 0 | 1 | 0 |
| C71.9 | Malignant neoplasm of brain, unspecified | 0 | 0 | 19 |
| C73 | Malignant neoplasm of thyroid gland | 0 | 1 | 1 |
| C74.9 | Malignant neoplasm of unspecified part of adrenal gland | 0 | 0 | 1 |
| C75.5 | Malignant neoplasm of aortic body and other paraganglia | 0 | 0 | 1 |
| C76.0 | Malignant neoplasm of head, face and neck | 0 | 0 | 1 |
| C76.2 | Malignant neoplasm of abdomen | 0 | 0 | 1 |
| C76.3 | Malignant neoplasm of pelvis | 0 | 1 | 0 |
| C77.9 | Secondary and unspecified malignant neoplasm of lymph node, unspecified | 1 | 0 | 2 |
| C78.0 | Secondary malignant neoplasm of lung | 1 | 0 | 10 |
| C78.1 | Secondary malignant neoplasm of mediastinum | 0 | 0 | 1 |
| C78.2 | Secondary malignant neoplasm of pleura | 0 | 0 | 2 |
| C78.6 | Secondary malignant neoplasm of retroperitoneum and peritoneum | 0 | 0 | 3 |
| C78.7 | Secondary malignant neoplasm of liver and intrahepatic bile duct | 1 | 0 | 18 |
| C78.8 | Secondary malignant neoplasm of other and unspecified digestive organs | 0 | 0 | 1 |
| C79.3 | Secondary malignant neoplasm of brain and cerebral meninges | 0 | 0 | 13 |
| C79.4 | Secondary malignant neoplasm of other and unspecified parts of nervous system | 0 | 0 | 1 |
| C79.5 | Secondary malignant neoplasm of bone and bone marrow | 0 | 0 | 6 |
| C79.8 | Secondary malignant neoplasm of other specified sites | 0 | 0 | 8 |
| C80 | Malignant neoplasm without specification of site | 0 | 0 | 11 |
| C81.9 | Hodgkin lymphoma, unspecified | 0 | 0 | 0 |
| C83.3 | Diffuse large B-cell lymphoma | 0 | 1 | 1 |
| C85.1 | Unspecified B-cell lymphoma | 0 | 0 | 1 |
| C85.9 | Non-Hodgkin lymphoma, unspecified | 0 | 0 | 9 |
| C90.0 | Multiple myeloma | 0 | 1 | 4 |
| C90.2 | Extramedullary plasmacytoma | 0 | 0 | 1 |
| C91.0 | Acute lymphoblastic leukemia [ALL] | 0 | 0 | 3 |
| C91.1 | Chronic lymphocytic leukemia of B-cell type | 0 | 0 | 3 |
| C92.0 | Acute myeloblastic leukemia | 0 | 0 | 3 |
| C92.1 | Chronic myeloid leukemia, BCR/ABL-positive | 0 | 0 | 1 |
| C92.4 | Acute promyelocytic leukemia | 0 | 0 | 1 |
| C97 | Malignant neoplasms of independent (primary) multiple sites | 0 | 0 | 1 |

**Supplemental Table 2.** Risk factors in childhood: association with subsequent cancer mortality additionally adjusted for family socioeconomic status

|  | n (cancer deaths) / N (number of observations) | Hazard ratio* | 95% CI* | | P |
| --- | --- | --- | --- | --- | --- |
| Systolic blood pressure | 173 / 13,366 | 1.03 | 0.89 | 1.20 | 0.67 |
| Diastolic blood pressure | 172 / 13,290 | 1.03 | 0.89 | 1.20 | 0.70 |
| Body mass index | 175 / 14,936 | 1.11 | 0.97 | 1.28 | 0.13 |
| LDL-cholesterol | 98 / 9,318 | 1.03 | 0.84 | 1.26 | 0.76 |
| Total cholesterol | 169 / 12,394 | 1.04 | 0.89 | 1.21 | 0.61 |
| Triglycerides | 169/12,393 | 1.01 | 0.86 | 1.17 | 0.94 |
| Smoking | 112/14,356 | 0.98 | 0.67 | 1.45 | 0.93 |
| Glucose | 73 /6,183 | 1.20 | 0.95 | 1.53 | 0.13 |

Continuous variables were standardized according to age, sex and cohort. Models were adjusted for age, sex, and cohort. Family socioeconomic status was assessed using information on maximum parental education and four categories were used (1=less than high school, 2=high school or equivalent, 3= more than high school/equivalent, 4= university degree).

* Per one standard deviation increase
